# A cross-sectional study of the temporal evolution of electricity consumption of six commercial buildings

**DOI:** 10.1371/journal.pone.0187129

**Published:** 2017-10-31

**Authors:** Ethan M. Pickering, Mohammad A. Hossain, Jack P. Mousseau, Rachel A. Swanson, Roger H. French, Alexis R. Abramson

**Affiliations:** 1 Department of Mechanical and Aerospace Engineering, Case School of Engineering, Case Western Reserve University, Cleveland, Ohio, United States of America; 2 Department of Electrical Engineering and Computer Science, Case School of Engineering, Case Western Reserve University, Cleveland, Ohio, United States of America; 3 Great Lakes Energy Institute, Case School of Engineering, Case Western Reserve University, Cleveland, Ohio, United States of America; 4 Department of Materials Science and Engineering, Case School of Engineering, Case Western Reserve University, Cleveland, Ohio, United States of America; 5 Solar Durability and Lifetime Extension Center, Case School of Engineering, Case Western Reserve University, Cleveland, Ohio, United States of America; Dalian University of Technology, CHINA

## Abstract

Current approaches to building efficiency diagnoses include conventional energy audit techniques that can be expensive and time consuming. In contrast, virtual energy audits of readily available 15-minute-interval building electricity consumption are being explored to provide quick, inexpensive, and useful insights into building operation characteristics. A cross sectional analysis of six buildings in two different climate zones provides methods for data cleaning, population-based building comparisons, and relationships (correlations) of weather and electricity consumption. Data cleaning methods have been developed to categorize and appropriately filter or correct anomalous data including outliers, missing data, and erroneous values (resulting in < 0.5% anomalies). The utility of a cross-sectional analysis of a sample set of building’s electricity consumption is found through comparisons of baseload, daily consumption variance, and energy use intensity. Correlations of weather and electricity consumption 15-minute interval datasets show important relationships for the heating and cooling seasons using computed correlations of a Time-Specific-Averaged-Ordered Variable (exterior temperature) and corresponding averaged variables (electricity consumption)(TSAOV method). The TSAOV method is unique as it introduces time of day as a third variable while also minimizing randomness in both correlated variables through averaging. This study found that many of the pair-wise linear correlation analyses lacked strong relationships, prompting the development of the new TSAOV method to uncover the causal relationship between electricity and weather. We conclude that a combination of varied HVAC system operations, building thermal mass, plug load use, and building set point temperatures are likely responsible for the poor correlations in the prior studies, while the correlation of time-specific-averaged-ordered temperature and corresponding averaged variables method developed herein adequately accounts for these issues and enables discovery of strong linear pair-wise correlation R values. TSAOV correlations lay the foundation for a new approach to building studies, that mitigates plug load interferences and identifies more accurate insights into weather-energy relationship for all building types. Over all six buildings analyzed the TSAOV method reported very significant average correlations per building of 0.94 to 0.82 in magnitude. Our rigorous statistics-based methods applied to 15-minute-interval electricity data further enables virtual energy audits of buildings to quickly and inexpensively inform energy savings measures.

## Introduction

In 2015, the U.S. commercial building sector accounted for approximately 8.7 quadrillion BTUs of energy consumption, 58% of which was electricity. This electricity accounts for approximately 37% of the total U.S. electricity consumption with 40% estimated to have been wasted, leaving a large opportunity for energy and cost savings in the building sector [1]. Studies have found that technologies available today may reduce energy use in commercial buildings by 30% and even as high as 55% considering further advancement of energy efficient technologies [2, 3]. However, implementing energy efficiency actions remains a challenge.

Energy audits, the identification and subsequent mitigation of energy efficiency losses, are essential in reducing commercial building energy consumption due to buildings’ long lifetimes and slow renewal rates [4]. However, commonly implemented energy audit methods and building information modeling can be costly, time consuming, and often provide uncertain results. Conventional energy audits require a team of individuals to perform walk-throughs of the building installing equipment,(e.g. occupancy sensors, equipment sub-meters, etc.),conducting various tests, and collecting answers to detailed questionnaires. Building Information Modeling (BIM) typically includes physics-based models (such as EnergyPlus, BLAST, DOE-2.1E, TRNSYS-TUD, and ESP-r) that require building managers to provide extensive detailed inputs regarding equipment data, floor plans, building materials, occupant schedules, etc. [5–9]. Both conventional energy audits and BIM have not achieved widespread adoption; each costs a significant amount of time and money, which can outweigh the identified energy savings. For these reasons, building managers frequently question the economic benefit of audits and can actively discourage their use [10–13].

Data science and analytics provides a promising alternative approach to conventional energy audits. [14–16] Due to advances in processing, data storage, communication, and analytics (such as distributed computing), it is becoming possible to use rigorous, data-driven approaches to uncover insights into building energy efficiency [17, 18]. Data analytics applied to buildings have been used to measure the energy savings associated with building retrofits [19, 20] and efficiency programs [21–24]. Data analytics provides two means of analysis: longitudinal studies of an individual building or cross-sectional studies of a sample set, or population, of buildings. Individual building analyses perform analytics on one building’s energy data, creating models and identifying potential energy-saving measures. Most of the current literature focuses on individual buildings, whether through conventional, BIM, or data analytic energy audit techniques [11, 20, 24, 25]. Population-based analyses, however, compare results from longitudinal building electricity usage studies between sample sets of several buildings in different climate zones to gain cross-sectional and comparative insights into energy efficiency by comparing and contrasting classes of buildings and climate zones due to differences and similarities across the buildings in the sample set being studied. The combination of both individual and population informed analyses can then lead to the understanding of variables previously overlooked [26]. One particular building energy population-based study looked at over 4000 residential buildings in Ireland, using 30-min interval time series datasets to determine methods for ranking energy efficiency relative to other buildings and consequently, prioritizing buildings for efficiency improvements [27]. Similar studies have yet to be conducted on commercial buildings. Considering the additional complexity of commercial versus residential buildings, a population-based analysis may yield further insights into building operation and opportunities for efficiency savings.

To date, most data analytics approaches to energy efficiency have used standard linear regression analysis. One of the first models, PRISM (PRInceton Scorekeeping Method), released in 1986, used regression analysis to measure energy savings in commercial buildings. [28] Utilities, companies, and government agencies used the statistical procedures of PRISM to analyze monthly utility bills to provide a weather-adjusted analysis of energy consumption before and after building retrofits. The U.S. Department of Energy’s EnergyStar Portfolio Manager built on the PRISM approach by including other datasets such as occupancy, plug load, and other variables and by developing a rating system for buildings associated with their energy efficiency [29]. ASHRAE also developed the Inverse Model Toolkit (IMT) for a similar purpose, [30] which was later extended to increase insight and accuracy [31, 30], and [32]. These data analytics approaches used low resolution energy data, such as yearly, monthly, or daily time intervals for linear regression models. However, standard regression models fail at finer temporal resolutions, such as hourly and sub-hourly datasets [33], and require much more complex methods for modeling [34]. Even daily resolution datasets require autocorrelation adjustments between energy and weather data to account for errors [16]. Studies have continued to analyze building data at hourly levels using regression analysis with only fair agreement to observed energy data, most likely due to the complex thermal relationship between weather and buildings [24, 35, 36]. In contrast, this work examines 15-minute interval electricity consumption paired with 30-minute and hourly weather datastreams to examine the relationships and correlations between weather and energy consumptions across six buildings. The analysis uncovers patterns, anomalies, and unique characteristics associated with specific buildings. This approach assumes that building information is inherently captured by historical and other readily available energy data. Consequently, building characteristics otherwise unidentifiable through standard means can be revealed.

In this paper we take an automated data analytics pipelining approach developed in *R* and will first describe our data science methods including data sources, acquisition, cleaning, assembly and analysis. We then present and discuss data-driven results of the cleaned datasets as well as the results of exploratory data analysis on a set of six building in two Koppen-Geiger (KG) climate zones [37, 38]. Further analysis is implemented on the relationship between electricity consumption and weather characteristics through a variety of methods, with the time-specific-averaged-ordered variable (exterior temperature) and corresponding averaged variables (electricity consumption) method shown to provide the most significant linear correlations. It is worth emphasizing this paper takes a data-driven modeling approach, as opposed to a physics-driven modeling approach, using first-order statistical methods to capture behavioral trends.

## Methods

**Fig 1:**
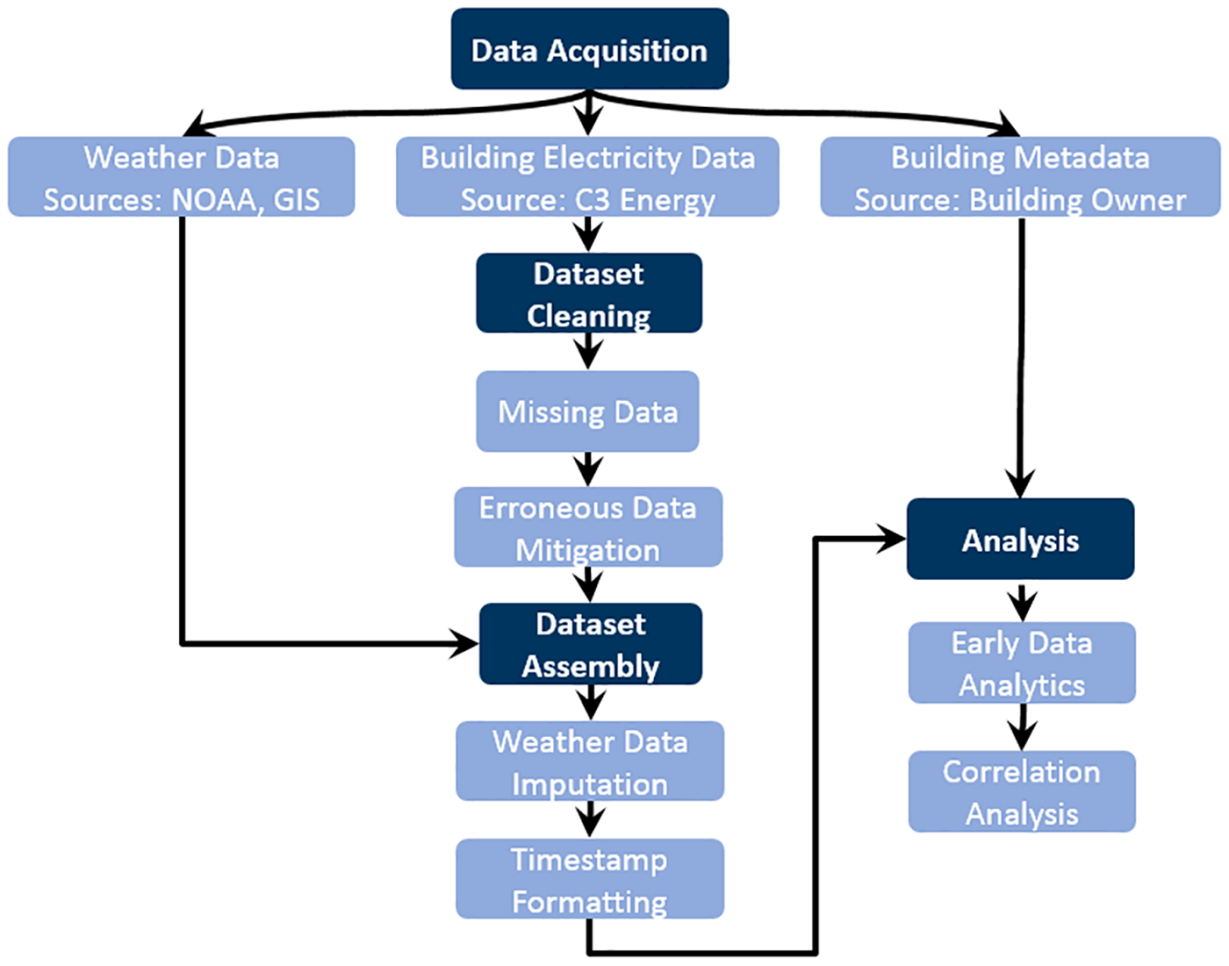
Data analysis flow chart. Data analysis flow chart of described methods and analyses.

Considering the vast amount of building energy time series datasets which are available to be analyzed, an open-data-science approach is taken using the software *R*. The *R* language is an open-source programming language developed for statistical computing, with a substantial community and more than 10,000 code packages available in the Comprehensive R Archive Network (CRAN) [39]. Packages beyond basic *R* installation implemented in this paper include: *RCurl* [40], *AnomalyDetection* [41], and *psych* [42]. The integrated development environment (IDE) *RStudio* was used extensively in the development of the code and visualization of the data [43]. The building energy and weather time series. datasets were acquired from National Oceanic and Atmospheric Administration (NOAA)noaa.gov, Solargis solargis.com, and C3 IoT c3iot.com. The sequential steps and methods used in our data analysis pipeline are presented and described by Fig 1.

### Data acquisition and sources

We analyze six commercial buildings in two different locations and KG climate zones: San Jose, California (Cfa) and Richardson, Texas (Csb). Table 1 describes each building by its location, size, type, climate classification [44], and heating, ventilation and air conditioning (HVAC) characteristics. These values were directly provided by the building manager and then stored as a JavaScript Object Notation (JSON) file accessible to the developed code. The building electricity consumption data were collected from revenue-grade utility electricity meters (kWh) taken at 15-minute intervals, measured as a function of time for approximately 2 years, and stored for the building owner by C3 IoT. The data were manually downloaded from their databases and saved as comma separated values(CSV) text files. Hourly weather data were collected from publicly available National Oceanic and Atmospheric Administration (NOAA) datasets (within 25 miles from building location) and privately held 30-minute interval Solargis (GIS) datasets (precise within 3.5 kilometers for each building location) were also employed [45]. The NOAA weather data were acquired for the NOAA location closest (and within 25 miles) to the building manager’s provided longitude/latitude from a public NOAA FTP endpoint. The data are fetched using the *RCurl* *R* package and then written to a CSV text file containing values of temperature, wind speed, and relative humidity. Meanwhile, the satellite-based geographic information system (GIS) weather data were ordered for the provided latitude/longitude and acquired as a CSV text file. The ground level GIS weather data were determined using an empirical atmospheric model produced by *SolarGIS* that utilizes satellite images and projects this information through the atmosphere to calculate ground-level temperature, wind speed, solar irradiance, and relative humidity. A comparison of the two datasets is performed in the results to determine discrepancies between the data and assess the quality of each.

**Table 1:** Building characteristics of all six buildings. Includes location, size, purpose, climate, and electric HVAC.

Building	Location	Size (*ft*^2^)	Purpose	Climate	Electric HVAC
Building 1	RN, TX	226000	Office/Lab	Cfa	Heat
Building 2	RN, TX	168000	Office/Lab	Cfa	Heat/Cool
Building 3	RN, TX	244000	Office/Lab	Cfa	Heat/Cool
Building 4	SJ, CA	109000	Office	Csb	Cool
Building 5	SJ, CA	115000	Office	Csb	Cool
Building 6	SJ, CA	168000	Office	Csb	Cool

The building electricity consumption data CSV contains two columns, local timestamp in local time and electricity consumption in MWh. Table 2 shows a sample of the raw data. The 15-minute interval energy consumption data were computed via time integration by the building owner. Over a 15-minute interval the power data, or load, of a building changes frequently; however, only the integration of the power load is reported for each interval. Even though this work was done with 15 minute interval data, we refer to timepoints for the time-series analysis instead of referring to datapoints by index number, since its important that the code is generalized for any time-series interval value.

**Table 2:** Example of raw building electricity data at six consecutive intervals.

Key.Performance.Indicators.	Utility.Electricity.Consumption..MWh.
2012-07-01T00:00:00-04:00	0.03785
2012-07-01T00:15:00-04:00	0.03677
2012-07-01T00:30:00-04:00	0.03727
2012-07-01T00:45:00-04:00	0.03551
2012-07-01T01:00:00-04:00	0.03605
2012-07-01T01:15:00-04:00	0.03695

### Dataset cleaning

Initial data validation was performed on all building electricity datasets to ensure the data quality was sufficient for further analyses. All datasets underwent visualization and preliminary statistical analysis to identify potential artifacts, outliers, and anomalies. In particular, the utility electricity data observed are partially incomplete, noisy, and anomalous, recording non-physical quantities or zeros at times. Using an anomaly detection package in *R* [41] developed by Twitter, anomalies were identified and then removed or corrected as necessary. Anomalies are defined in this context as instances in the data which are either erroneous or unusual, but not necessarily incorrect (i.e. higher usage for a sustained period of time). This analysis determines when three distinct types of anomalies are present: extreme outliers, energy shifts, and missing data. Here extreme outliers are defined as values an order of magnitude above the mean electricity usage of the entire dataset:

$$\begin{array}{c} Outlier\geq \frac{10}{n}\times \sum_{i=1}^{n}E_{i} \end{array}$$ (1)

where *E*_*i*_ is the electricity consumption in kWh at any timepoint and *n* is the number of timepoints in the dataset. Once identified these values were simply removed and tagged. The second anomalies are energy shifts, which occur when the electricity consumption data has moved from one consistently observed average energy value to a new and distinctly different averaged energy value. These occurrences were detected using two four-day moving averages computed along the electricity data (eight total days analyzed at once) represented as *MA*_1_ and *MA*_2_ given by:

$$\begin{array}{c} MA_{1}=\sum_{i}^{i+96hrs}E_{i} \end{array}$$ (2)

$$\begin{array}{c} MA_{2}=\sum_{i+96hrs}^{i+192hrs}E_{i} \end{array}$$ (3)

When

$$\begin{array}{c} n-0.2\times MA_{1}<MA_{2}<n+0.2\times MA_{1} \end{array}$$ (4)

where *n* is any integer value, an energy shift is identified at *i* + 96*hrs* until

$$\begin{array}{c} MA_{2}<fracMA_{1}n-0.2 \end{array}$$ (5)

when the energy shift is seen to return to original values. The purpose of this method is primarily to identify instances of integer multiple counting of the data such as double counting. The confidence interval of ±0.2 from any integer value was used to account for other fluctuations in the data not due to the energy shift. Therefore any shift in the data within an integer multiple ±0.2 is considered an energy shift and consequently is determined to have been multiply counted by a meter (e.g. the range 1.8−2.2 × *MA*_1_ returns an integer multiple of 2 or a range of 2.8−3.2 × *MA*_1_ returns 3). Using this information the electricity data can be adequately corrected before analysis. For the case of double counting, the identified data points were simply divided by 2. However, if the data contained energy shifts of a non-integer multiple no correction was taken. The third anomalies addressed were missing data points in electricity consumption. Here, imputation of missing data points by linear interpolation for intervals less than one hour was used; in this research imputed electricity data were < 0.1% of the total dataset. In the event that a meter lost connection for this short interval, the rest of the day’s electricity usage is still worthy of analysis. This step allowed additional days to be analyzed, despite minor data losses. Those days which exhibit data losses at an hour or larger were not imputed to minimize inaccurate conclusions. All cleaned data for the six building datasets reported anomalies of less than 0.5%.

### Dataset assembly

The electricity, NOAA, and GIS data files were acquired in various formats, requiring scripted processing to produce a uniform structured dataframe for analysis. Timestamps for all datasets (NOAA, GIS, and electricity) were formatted to POSIX, (which uses the number of seconds from the beginning of 1970 in the universal coordinated time zone (UTC)), and set to the local time of each building. However, the timestamps from each dataset did not perfectly align. The NOAA datasets provided only hourly weather data, while GIS has 30-minute interval datasets, and building electricity is in 15-minute intervals datasets. To account for the differences in timestamps the weather data (NOAA and GIS) were imputed by linear interpolation at the 15-minute intervals corresponding to the building electricity data. This linear interpolation was used since weather characteristics (e.g. temperature) do not vary drastically between hourly or sub-hourly intervals. The datastreams from all three data sources were assembled into an R dataframe for each building, with predictors (independent variables) and responses (dependent variables) as columns, and the observations stored as rows, index by their 15-minute timestamp. Additionally, due to the use of local time, daylight savings days were omitted from the analysis as non-24 hour days posed problems in analysis.

### Exploratory data analyses methods

Once the data were cleaned and processed, further exploratory data analyses (EDA) could be implemented. EDA provides a means to determine the basic characteristics of, and relationships between, the variables in the building datasets using both quantitative methods and data visualization. In this section quantitative measures such as comparisons of electricity usage and the correlations of electricity consumption and weather, and visual EDA are shown for the datasets and pair-wise univariate relationships.

#### Building comparisons

The energy data alone were assessed for baseload, daily variation, percentage of daily variation to baseload, and the energy use intensity (EUI). The baseload, *B*, was computed as the 5th percentile of minimum energy values of each day, rather than the absolute minimum value of energy, and is given by

$$\begin{array}{c} B=E_{min,i}. \end{array}$$ (6)

*E*_*min*_ is the subset of values containing the minimum values of each day

$$\begin{array}{c} E_{min}\subset {\left(\min E_{j=1}^{n}\right)}_{k=1}^{N} \end{array}$$ (7)

and then sorted in ascending order, where *j* = 1: *n* represents the data points of an entire day while *k* = 1: *N* denotes the amount of days in the dataset. The index, *i*, in Eq 6 is the index of the 5th percentile given by

$$i=\left\lceil \frac{5N}{100}\right\rceil$$ (8)

where *N* is the number of days in the analysis. This method was chosen to minimize the effect of any erroneous data which may have passed through cleaning and result in incorrect baseload values when taking the absolute minimum. The daily variation, *DV* was determined by averaging day energy ranges given by

$$\begin{array}{c} DV=\frac{1}{N}\sum_{i=1}^{N}E_{range} \end{array}$$ (9)

where

$$\begin{array}{c} E_{range}\subset {\left(\max E_{j=1}^{n}-\min E_{j=1}^{n}\right)}_{k=1}^{N}. \end{array}$$ (10)

Finally, the energy use intensity, *EUI* was calculated as

$$\begin{array}{c} EUI=\frac{1}{T\times A}\sum_{i=1}^{m}E_{i} \end{array}$$ (11)

where *T* is in years, *A* is the building squarefootage, *m* is the number of data points, and *E* is the energy in *kBtu*. This equation results in *EUI* of units *kBtu*/*ft*^2^ × *yr* as is standard of U.S. Department of Energy EUI databases [46]. However, note that this analysis only included utility electricity data and not other sources of energy such as natural gas, and therefore cannot be interpreted as a conventional EUI assessment.

#### Correlations between weather and energy

For most buildings, the largest fraction of energy usage comes from HVAC [47], which is correlated with the outdoor weather characteristics. Therefore using NOAA and GIS weather datastreams to understand the relationship between building energy use and weather is critical, since correlations and scatter plots of electricity versus weather data reveal these relationships. Pair-wise correlation scatter plots were created, using the function panel.pairs() from the *R* package *psych* [42], which show the Pearson linear correlation between all variable pairs in the dataset. Linear correlations are used to identify the first-order correlation between variables and whether that relationship is negative or positive. With the insight gained from these linear correlations, further and more complex models can be developed to investigate the potential existence of higher order correlations among these parameters. In this analysis, the variables analyzed were: electricity consumption (*kWh*), exterior temperature (NOAA (*°F*) and GIS (*°C*)), and irradiance (global horizontal irradiance (GHI - *W*/*m*^2^)). Relative humidity correlations were ∼ 0 for all buildings and were excluded from the analysis. Irradiance was included in this analysis as it effects the thermal load of a building [7, 48].

To improve the correlation analysis, heating and cooling periods were analyzed separately using heating/cooling degree day (HDD/CDD) temperature of 65*°F* (18.3*°C*) as the delineator [49]. To ensure days were strictly heating or cooling operations, an offset of the base temperature was defined for each individual climate, as each location undergoes varying temperature fluctuations (i.e. San Jose experiences mild weather with minimal daily fluctuations, while Richardson experiences more extreme weather with larger daily fluctuations). The offset was defined as half of the standard deviation of the day mean temperatures, *T*_*mean*_, with heating operations as

$$\begin{array}{c} T_{mean}<65^{o}F-\sigma /2 \end{array}$$ (12)

and cooling operations

$$\begin{array}{c} T_{mean}<65^{o}F-\sigma /2 \end{array}$$ (13)

where

$$\begin{array}{c} T_{mean}\subset {\left(\sum_{j=1}^{n}E_{i}\right)}_{k=1}^{N} \end{array}$$ (14)

and *σ* is the standard deviation of mean day temperatures, *T*_*mean*_. The mean temperature (NOAA) and standard deviation for Richardson were 67.3*°F* and 16.9*°F* and for San Jose 59.7*°F* and 7.1*°F*. This analysis also provides an opportunity to determine the general type of heating and cooling systems (e.g. electric, gas, district energy). For example, during the heating season, a strong negative correlation between electricity consumption and exterior temperature indicates the presence of an electric heating system (i.e. when temperature decreases, consumption increases).

Finally, two more approaches were taken to determine the correlation coefficients. As previously mentioned, a significant amount of a building’s electricity consumption (e.g. such as occupancy and plug load) is not a result of weather changes. One approach to minimize these loads is to assume behavior-induced electricity consumption occurs similarly at a given time each day, and then hold each time of the day constant in analysis. That is, only compare one time of the day against the same time for all days in the dataset(e.g. all 9 a.m. values compared with only 9 a.m. values). This was done by subsetting the datasets for each time of the day and taking the correlation between the electricity and temperature values. The subsets are defined for electricity and temperature as

$$\begin{array}{c} \begin{array}{c} E_{timej}\subset {\left(E_{j}\right)}_{k=1}^{N}\\ T_{timej}\subset {\left(T_{j}\right)}_{k=1}^{N} \end{array} \end{array}$$ (15)

where *k*: *N* represent all days in the analysis and *j* refers to the time point of the day. Both the electricity and temperature subsets are created for each time of the day and are correlated as such

$$\begin{array}{c} C_{j=1}^{n}={\left(cor\left(E_{timej},T_{timej}\right)\right)}_{j=1}^{n}. \end{array}$$ (16)

Therefore reporting a vector of the linear correlations of the *n* time points within a day of data. In this analysis, one day consists of 96 timepoints and consequently 96 correlations (i.e. 24 hours at 15-minute intervals gives 96 data points per day). This analysis is referred to as the time-specific correlation method.

Furthermore, the time-specific correlation method can be modified to additionally minimize the occupancy and plug load interference. The previous analysis assumes occupancy and plug load contributions are relatively constant for all days; however, the uncertainties of human occupant behavior are a major challenge in predicting building energy, making this previous assumption extremely weak [50]. To minimize this uncertainty/randomness, each time-specific dataset (both electricity and temperature) was reordered by temperature from highest to lowest resulting in $E_{timej}^{\prime }$ and $T_{timej}^{\prime }$. Using this new order, groups of 30+ observations (30 days at each specific time) of most similar temperatures were determined and averaged within each time point

$$\begin{array}{c} \begin{array}{c} E_{avg.timej,l}^{p}=\frac{1}{30}[\sum_{i=1}^{30}E_{timej,i},\sum_{i=31}^{60}E_{timej,i},...\frac{30}{N-(30(p-1)+1)}\sum_{i=30(p-1)+1}^{N}E_{timej,i}]\\ T_{avg.timej,l}^{p}=\frac{1}{30}[\sum_{i=1}^{30}T_{timej,i},\sum_{i=31}^{60}T_{timej,i},...\frac{30}{N-(30(p-1)+1)}\sum_{i=30(p-1)+1}^{N}T_{timej,i}]. \end{array} \end{array}$$ (17)

Then correlating each time-specific set of vectors resulted in a new correlation vector C:

$$\begin{array}{c} C_{l=1}^{p}={\left(cor\left(E_{avg.timej},T_{avg.timej}\right)\right)}_{l=1}^{p}. \end{array}$$ (18)

This method is called the correlations of a time-specific-averaged-ordered variable and the corresponding averaged variable (TSAOV), where temperature and electricity are the variables respectively. By grouping at least 30 observations within each time-specific subset with similar temperature values, the average, or typical, occupancy and plug load value was able to be held constant through a range of temperatures while also maintaining, or even refining, the response of building electricity to temperature.

## Results and discussion

### Data cleansing

Three types of anomalies were found throughout the datasets: outliers, missing data, and energy shifts and each of these anomalies can impede robust analysis and graphical representation. Outliers are relatively simple to identify and omit, but missing data and mean shifts present larger analysis issues. Missing data skew the analysis and requires the omission of those time stamps when gaps are larger than an hour—resulting in the omission of months of data that could have been useful in the analysis. Energy shifts are the result of meter error which does not require the elimination of data, but does require adjustment before analysis.

**Fig 2:**
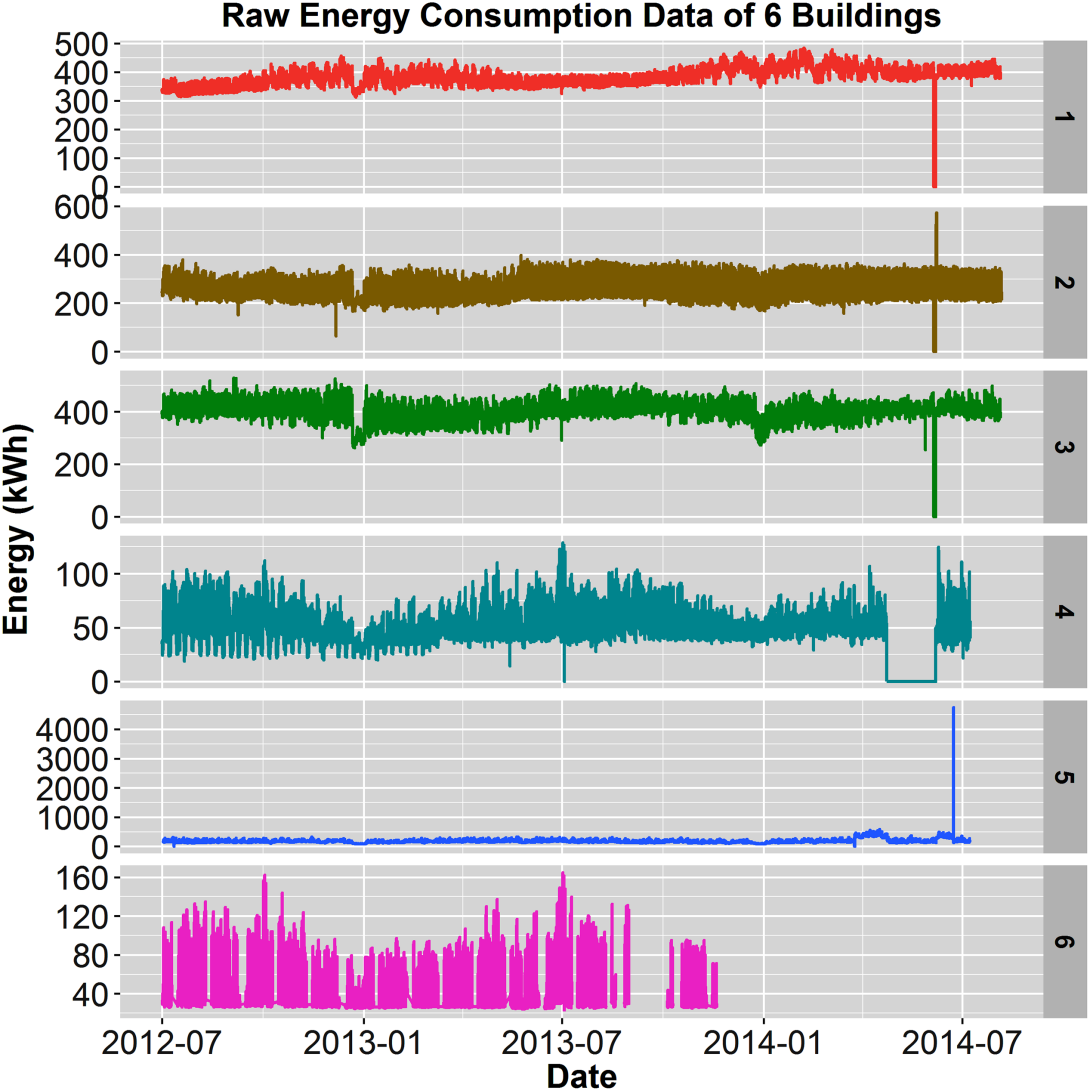
Raw electricity consumption. Raw electricity consumption data from full datasets for all 6 buildings(building number shown on the right most side of each plot). Building 5 shows the presence of outliers and mean-shifts; all buildings exhibit missing data.

**Fig 3:**
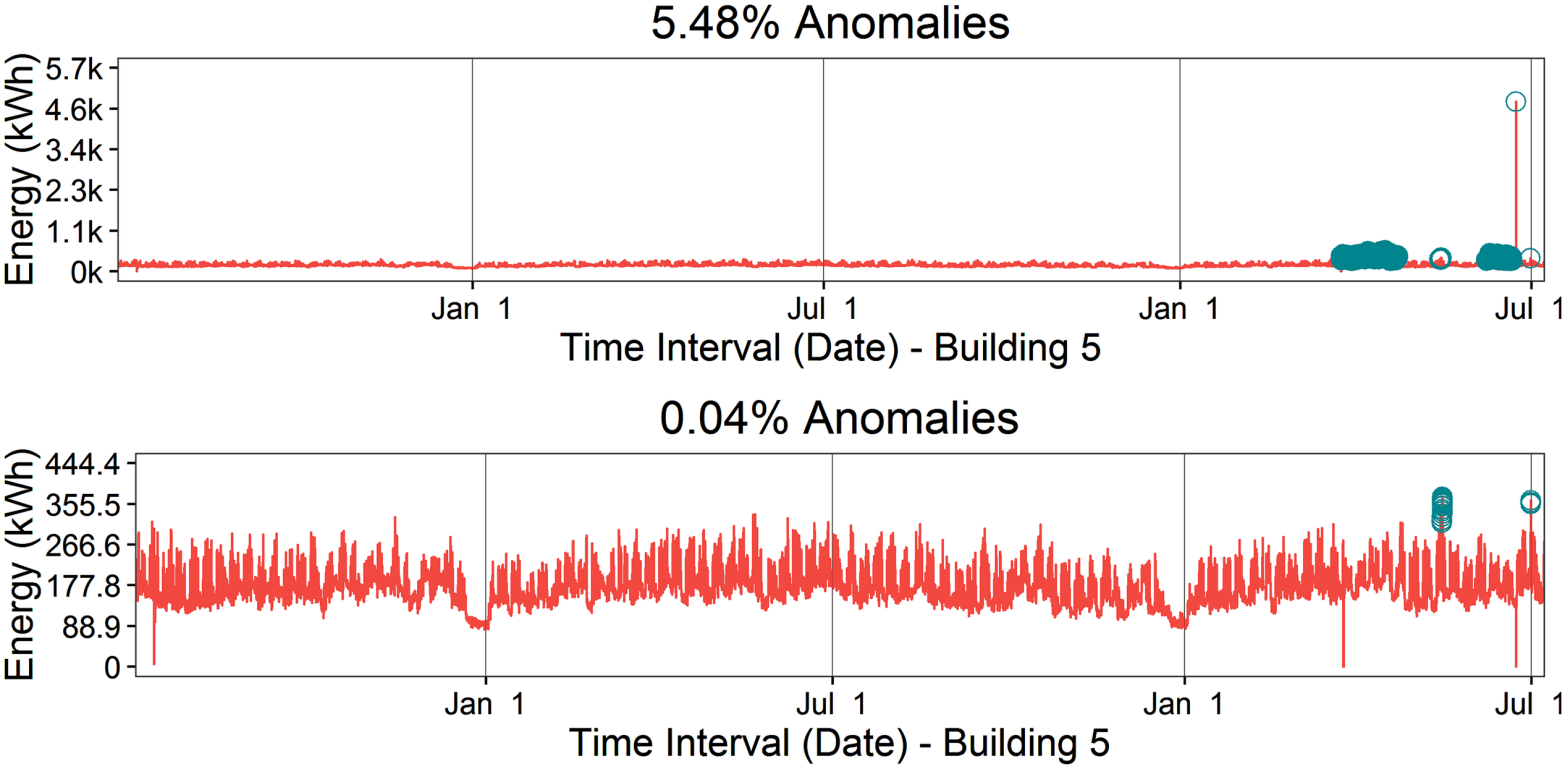
Electricity consumption anomaly cleaning. Building 5(top) raw electricity consumption data (5.48% anomalies) and (bottom) cleaned electricity consumption data (0.04% anomalies).

**Fig 4:**
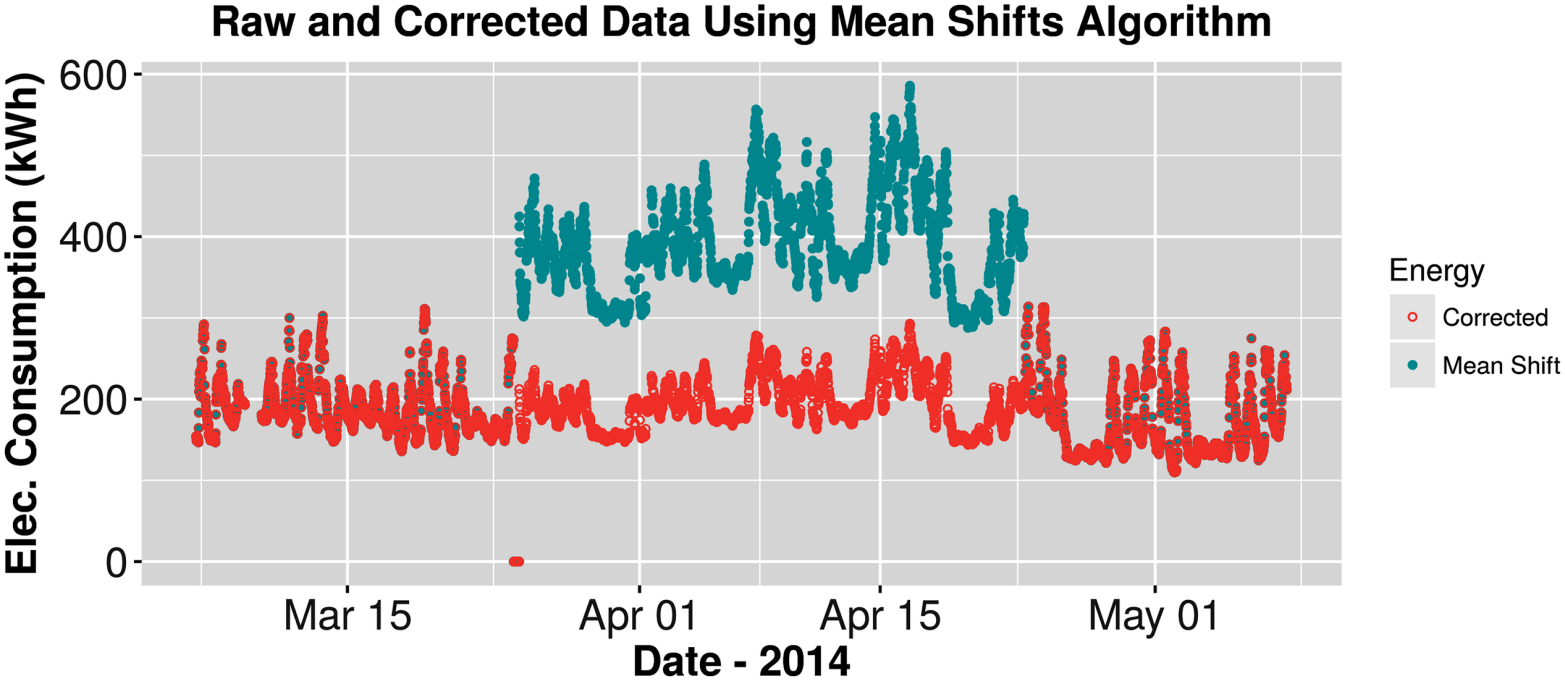
Mean shifts. Nine week view of Building 5 mean shifts. Original data displayed as shaded blue circles and corrected data as red hollow circles.

Fig 2 displays the raw electricity consumption for the six buildings over two years, 2012-2014, with the exception of Building 6, which only 1.25 years was available. All buildings show various anomalies and, in particular, Building 5 shows extreme outliers and energy shifts that render the visual useless. Those anomalies include a large spike (outlier) toward the end of the dataset, as well as two raised sections of electricity consumption (energy shifts). Fig 3 shows Building 5 with 5.48% anomalies before cleaning and with 0.04% anomalies following the handling of missing data, outliers, and energy shift correction. Additionally, Fig 4 displays a closer view of Building 5’s energy shift before and after data cleaning. Note that the difference between the raw and cleaned energy consumption data for Building 5 is 814 MWh, which is equivalent to $155,000 at $0.19/kWh. In other words, meter error alone may have led to unnecessary utility charges, in this case an over-billing of $155,000.

**Fig 5:**
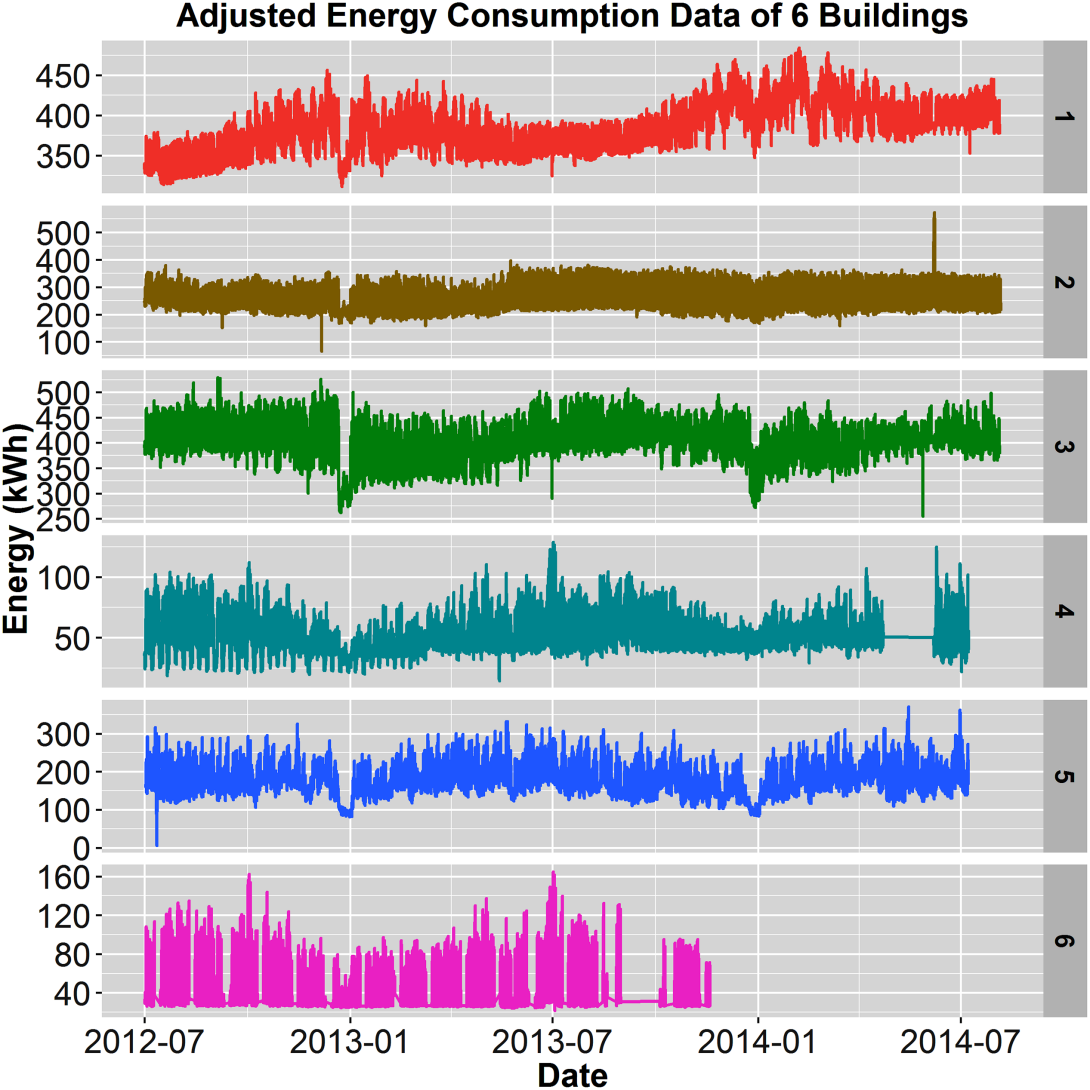
Cleaned electricity data. Cleaned electricity consumption of full datasets for all 6 buildings.

All datasets were cleaned using the methods described early and Fig 5 shows all six buildings after passing through data cleaning and assembly. These results show the importance of data cleaning in providing correct data and mitigating anomalies.

### Exploratory data analytics

#### Building comparisons

**Fig 6:**
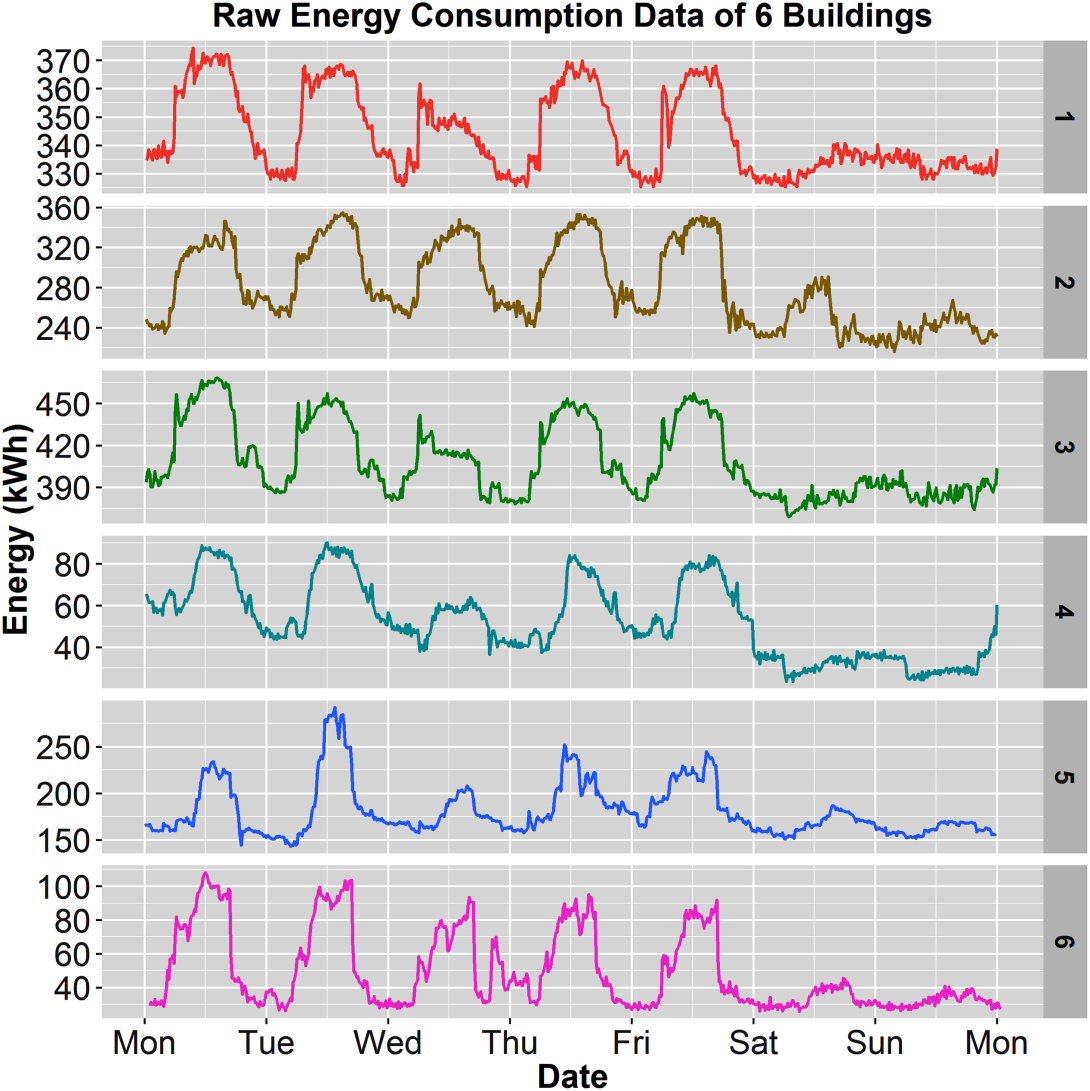
Week view of electricity consumption. Electricity consumption of one week for all 6 buildings. Building number label is to the right of each plot.

The cleaned energy datasets were then analyzed to assess baseload, daily variation, percentage of daily variation compared to baseload, and energy use intensity. The analysis is represented in Table 3, and can also be compared to Figs 5 and 6. Comparing the baseload between the 6 buildings in Table 3, those with lab spaces reported very high baseloads, such as buildings 1, 2, 3, and 5, while office-only spaces exhibited much smaller baseloads by 4-13 times. Even partial lab buildings (Building 1 and 3) presented baseload values almost 3 times the size (300kWh) of the smallest lab (Building 5 at 100kWh). In comparing EUI values of all four building, labs fell between 180-202 *kBTU*/(*ft*^2^ × *yr*) which are consistent with the Department of Energy’s Portfolio Manager for lab space [46]. However, considering these values omit non-electric HVAC, building EUI is likely much higher, indicating room for efficiency savings. The office buildings, located in San Jose, CA, showed a much smaller EUI (avg. EUI of 90.8) than those in Richardson, TX (195.1). Climate differences among these locations is the most likely factor for this large difference in EUI. Finally, the daily variance and percentage of daily variance to baseload varied wildly among the six buildings from 12.6%-213% presented in Table 3 and shown graphically in Fig 5, as well as in Fig 6. This metric, percentage of the daily variance, is important as it allows one to define the relative impact these analyses could have on identifying efficiency problems associated with a building’s operation.

**Table 3:** Baseload, daily variance, percentage of daily variance to baseload, and energy use intensity (electricity consumption only).

Building	Baseload	Daily Variance	Daily/Base	EUI
	kWh	kWh	%	$\frac{kBtu}{ft^{2}\times yr}$
Building 1	324.90	40.86	12.58	201.95
Building 2	182.25	110.10	60.41	188.11
Building 3	316.35	73.12	23.11	195.36
Building 4	23.99	32.86	136.98	58.57
Building 5	108.24	76.11	70.31	180.38
Building 6	24.98	53.19	212.96	33.34

Buildings with large baseloads (> 100*kWh*) and otherwise small daily variations (< 75% of the baseload) may not allow for significant enough variability which is necessary in order to uncover specific issues. Nonetheless, those buildings with high baseloads may indicate the presence of systems or equipment that should be operating variably and not as part of the baseload (e.g. requiring setpoint changes at night or during periods of low occupancy). For example, Building 1 from Table 3 exhibits a 15-minute baseload consumption of 325kWh with 41kWh of daily variation, so only 11% of the consumption may be probed for further analysis. Conversely, Building 6 exhibits a daily variability of 68%, which through further analysis may reveal markers associated with HVAC scheduling, large equipment usage, lighting consumption, and envelope insulative value among others not currently considered. Therefore, the comparison of these six buildings demonstrates the utility and potential for a population-based analysis for virtual energy insights when applied to much larger building databases.

#### 0.0.1 Weather data

**Fig 7:**
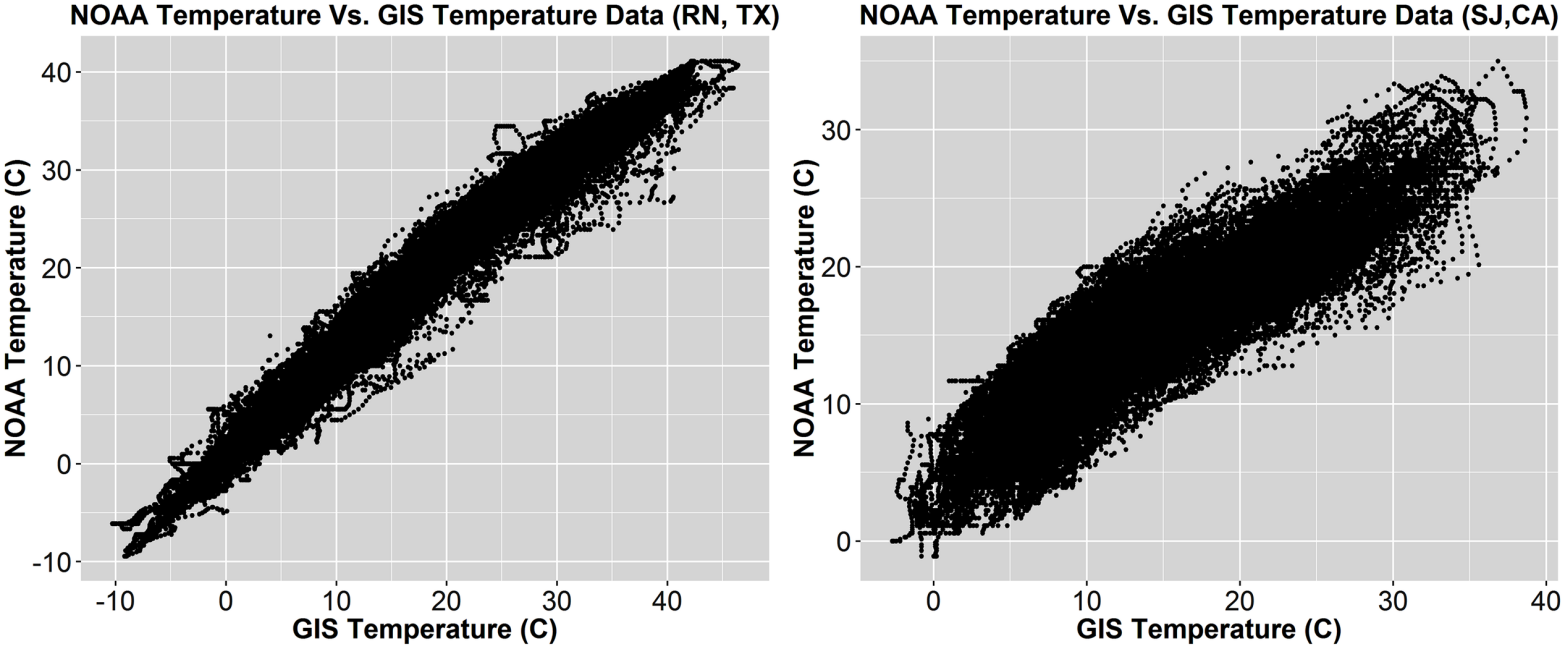
Comparison of NOAA and GIS temperature data (*°C*). Left: Richardson, TX (r = 0.995), Right: San Jose, CA (r = 0.887).

Both NOAA and GIS-derived weather datasets were used in this analysis to determine discrepancies between the weather data and assess quality of each. The differences between GIS and NOAA data include sampling rate, location accuracy, and measurement techniques. The GIS datasets provide 30-minute intervals, are accurate within 3.5km, and the data are computed using an atmospheric model, while the NOAA datasets include hour intervals, are accurate to at least 25 miles (although often less), and are recorded via direct sensors. GIS datasets prove optimal in both sampling rate and location accuracy, however, NOAA data has a higher accuracy when considering their use of ground-based sensors (measuring temperature, wind speed, and relative humidity). Comparisons between temperature datasets result in a Pearson linear correlation coefficient R = 0.995 for Richardson, TX and R = 0.887 for San Jose, CA as shown in Fig 7.

Considering these strong correlation coefficients, the data are relatively indifferent. This indicates the use of either dataset as valid for the analyses performed in this paper. However, it is worth noting the lower coefficient in San Jose. This is most likely due to the large climate variability associated with the region in the San Francisco Bay area, whereas Richarson’s larger correlation can be attributed to the Dallas area’s homogeneous climate. Therefore, although both datasets are similar and valid for analysis, locations with larger climate variability should favor GIS datasets over NOAA. One additional note between the datasets is price and inclusion of irradiance. NOAA is readily available, entirely free, and open sourced, while GIS is also readily available, but must be purchased and includes irradiance.

#### Correlations of weather and energy

**Fig 8:**
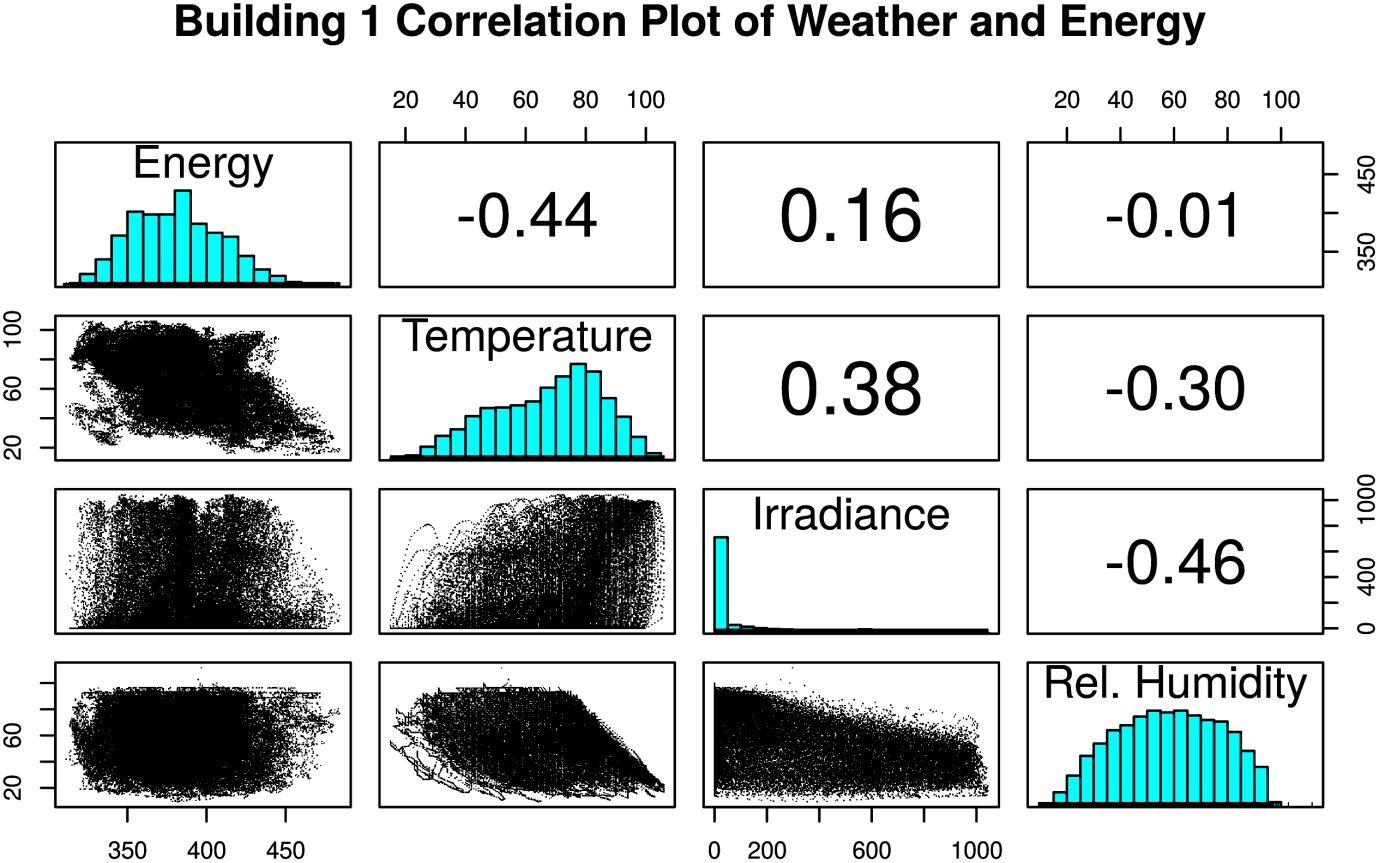
Correlations of electricity consumption and weather. Correlation plot of weather data and energy for Building 1.

Weather is known to impact the electricity consumption of buildings and consequently significant correlations were expected between weather data such as temperature and solar irradiation to electricity consumption. Fig 8 shows a pair-wise correlation plot corresponding to Building 1’s full dataset, with direct correlation coefficients of energy to exterior temperature of -0.44 (GIS), and energy to irradiance of 0.16. All three of these correlations are surprisingly low, representing a weak to moderate (less than 0.67 magnitude [51]) negative correlation. The negative correlation coefficient associated with Building 1 indicates that the building electricity load increases with decreasing temperature, leading to the probability that electric (or partial electric) heating systems are present in the building, although electric cooling is not. Table 4 displays all of the correlations among all six buildings using full datasets. The relatively weak correlation coefficients (< 0.67) found in this analysis indicates that other factors in the building must be influencing the correlation.

**Table 4:** Correlations for all 6 buildings using NOAA/GIS exterior temperature and energy consumption full datasets.

Building	NOAA Temp.	GIS Temp.	Irradiance
Building 1	-0.44	-0.44	0.16
Building 2	0.27	0.29	0.50
Building 3	0.40	0.42	0.38
Building 4	0.62	0.62	0.52
Building 5	0.53	0.52	0.51
Building 6	0.07	0.60	0.67

To further analyze these univariate linear correlations, the data were split into heating and cooling seasons. During the heating season, increasing electricity consumption should correlate with decreasing exterior temperatures to maintain indoor temperature (a negative correlation), while during the cooling season an increase in energy consumption should correlate to an increase in exterior temperature (a positive correlation), assuming electric systems are used for heating and cooling respectively. As shown in Table 5, in Building 1, the temperature correlations from the subsetted cooling-season data (exterior temperatures above 65*°F*) are essentially uncorrelated (-0.06 and -0.03) as expected since this building does not use an electric cooling system (Table 1). From the heating-season data (temperatures below 65*°F*) correlations are -0.33 and -0.38, which point to the presence of some electric systems used for heating, albeit the correlations are still rather weak.

**Table 5:** Correlations of energy consumption and weather variables for all 6 buildings using NOAA and GIS datasets. The left three columns correspond to the subsetted cooling data while the right three columns correspond to the heating season.

Building	Cooling Operation			Heating Operation		
	NOAA Temp.	GIS Temp.	Irradiance	NOAA Temp.	GIS Temp.	Irradiance
Building 1	-0.06	-0.03	0.36	-0.33	-0.38	0.24
Building 2	0.28	0.30	0.52	-0.04	-0.01	0.40
Building 3	0.34	0.35	0.41	0.06	0.07	0.22
Building 4	0.45	0.41	0.29	0.38	0.35	0.09
Building 5	0.34	0.25	0.28	0.36	0.33	0.32
Building 6	0.04	0.31	0.38	0.05	0.36	0.58

The remaining five building correlations can be compared to the HVAC system characteristics from Table 1. Both Building 2 and 3 possess heating and cooling electric HVAC systems even though their correlations for cooling are positive nonzero values (i.e. they agree with expected results), heating operation values are essentially zero when they should reflect negative nonzero values. Buildings 4 and 5 provide electric cooling only, yet they exhibit similarly strong positive correlations with temperature for heating. Finally, Building 6, an electric cooling only building, presents a third misrepresentation, as the data does not indicate a significant difference in correlations between cooling and heating operations. Considering five of six building’s correlations differ from expected results, simply subsetting the data into heating and cooling seasons does not alone lead to expected correlations between HVAC electricity consumption and exterior temperature. These poor correlations may be the result of a natural thermal lag of the building (due to thermal mass) and/or results from occupancy or other operational variations in the building, which are both addressed in the final approaches.

#### Influence of thermal mass and occupancy

**Fig 9:**
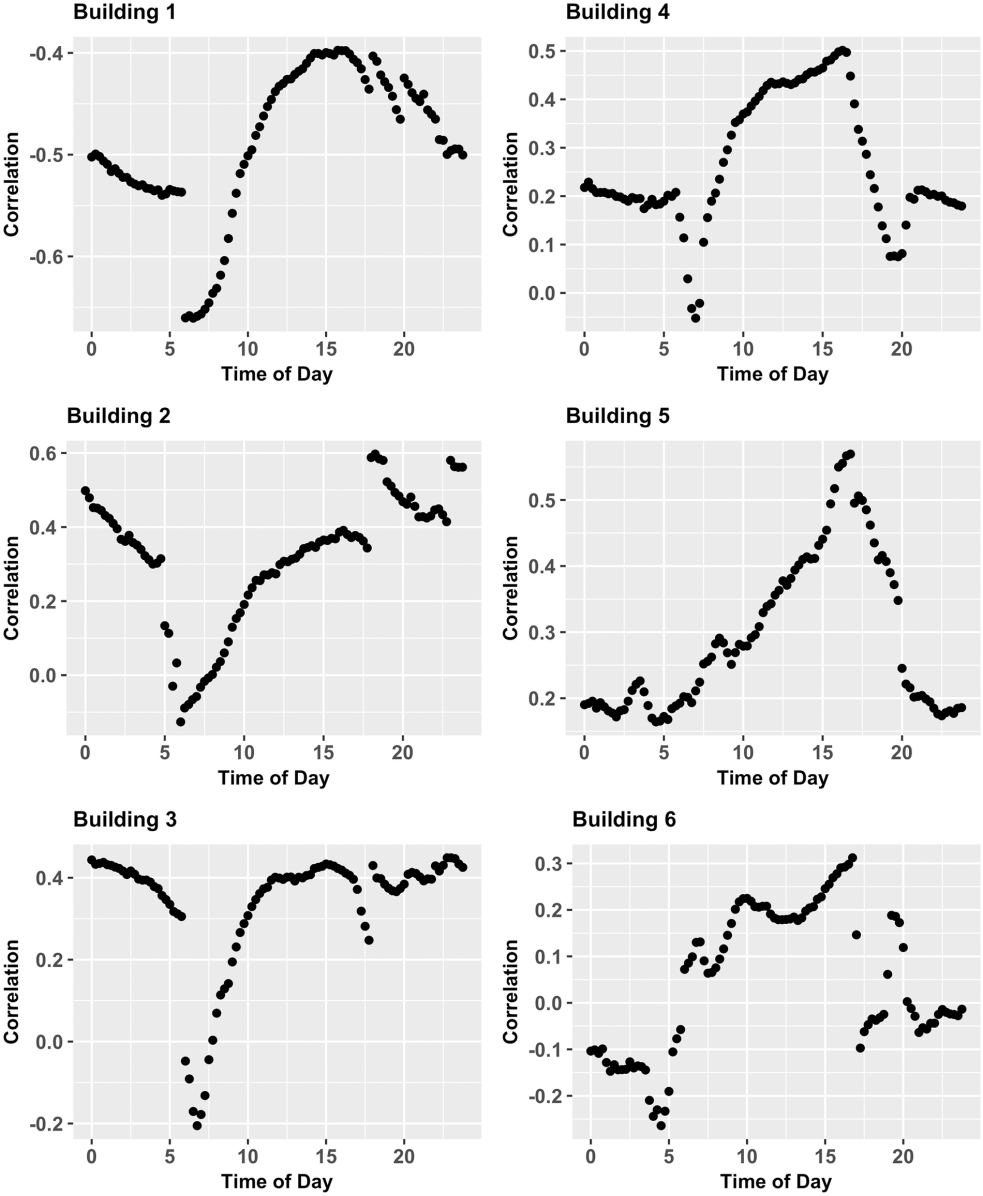
Correlations of time-specific variables. Correlation of time-specific variables, electricity consumption and temperature, for all six buildings.

Two approaches were taken to examine the influence of thermal mass and occupancy or other operational variations in the building. The first approach, correlations of time-specific variables, examined the resulting correlations when only specific times of the day are compared against one another. For example, each time of the day results in its own correlation, 6 am data are only correlated with 6 am data, or 9 am with 9 am, and this is done to mitigate the effects of operational tendencies (such as interior lighting turning on at 6 am each day) in a building. Therefore, the result of this analysis is *n* separate correlations, one for each time of the day. Fig 9 displays the correlations for each time of the day on all 6 buildings. All six plots show low correlation coefficients between electricity consumption and temperature. The largest correlation coefficient among all the pairs is -0.66, which is significant; however, virtually all the remaining values have magnitudes below 0.5. These poor correlations are still attributed to interference between occupancy and plug loads, which are largely mitigated in the following TSAOV method of analysis.

Despite the low values, there still remain striking findings in these plots: in particular, the variability of the correlations throughout the day. For all buildings, with the exception of Building 5, large drops are seen in correlation values during the early morning around 6 a.m. This is attributed to building startup times, which are scheduled events and not temperature induced. Similar behavior can be seen in the later hours in many of the buildings where the correlation either rises or drops suddenly. The difference in rising or dropping during evening hours is unclear, but may indicate a relationship between a building’s occupied to unoccupied set point change in the evening. Further investigation could lead to the determination of a building’s thermal mass by observing the correlation recovery time after an evening shutdown.

#### Time-specific-averaged-ordered variable method for weather correlations

**Fig 10:**
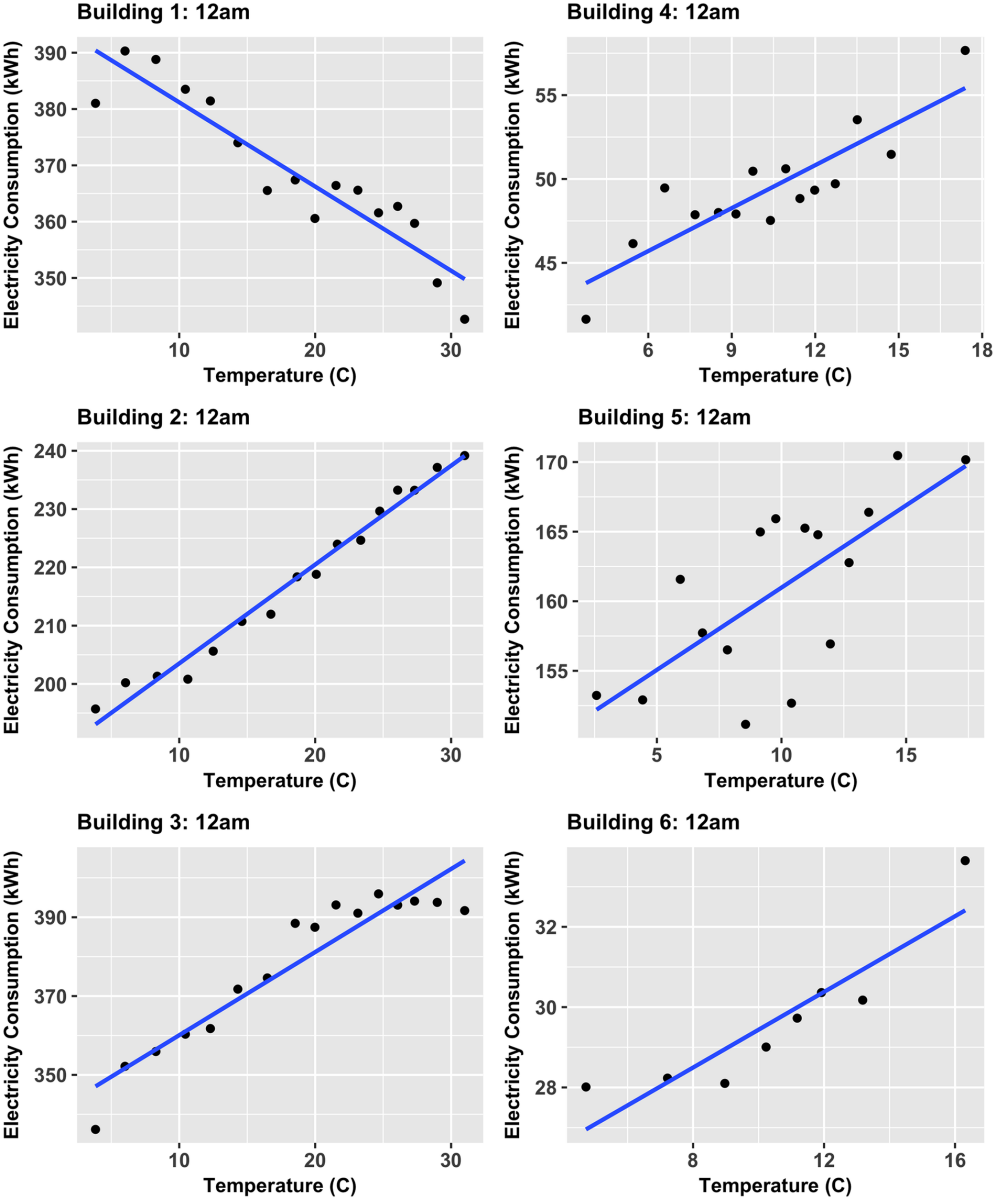
TSAOV method of temperature and electricity consumption for 12am. TSAOV-computed average electricity consumption against averaged outside temerpature for all buildings at 12am.

**Fig 11:**
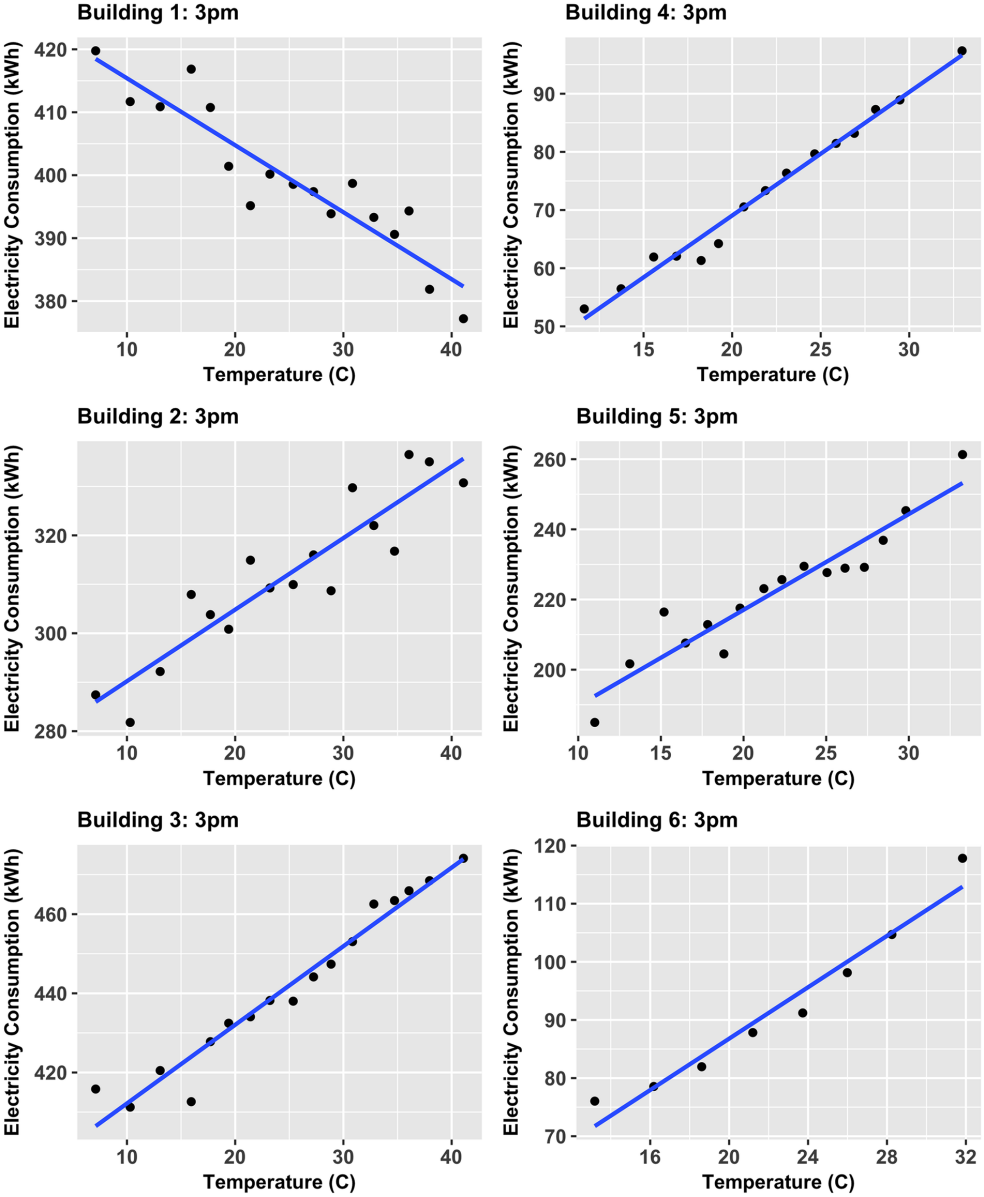
TSAOV method of temperature and electricity consumption for 3pm. TSAOV-computed average electricity consumption against averaged outside temerpature for all buildings at 3pm.

To account for the poor overall correlation values, the correlations of a time-specific-averaged-ordered variable and corresponding averaged variable method was implemented. The TSAOV method takes two more steps from the previous analysis by grouping the data (already subsetted by each individual time of day) by temperature in groups of 30+ observations (i.e. 30 days at a specific time) of most similar temperature. Then the mean temperature and electricity were computed for each group and correlations were calculated across groups of the same time of day. To further demonstrate the data-driven linear correlation approach taken, two TSAOV-computed datasets of the 96 times, 12am and 3pm, are shown accompanied with a linear best fit in Figs 10 and 11 respectively. Note that in these figures, each data point is representative of the average electricity consumption of 30+ observations at similar temperatures and plotted against the average temperature of those similar temperatures. The 3pm data show strong linear trends among all buildings, indicating linear correlations are the optimal choice for analyzing the temperature-electricity relationship. One could argue that Building 6 shows a weak nonlinear trend, however the linear approximation still closely captures the behavior. Further, the 12am times for all buildings, except for Building 5 (which still shows a generally positive linear trend at this particular time), also exhibit strong linear trends. When considering these 12 comparisons, it is evident that a linear correlation analysis presents the most general and universally accurate method to compare multiple buildings. The use of linear correlations here are also a result of many of the six buildings having either electric heating or cooling, but not both, meaning electricity should respond either monotonically negative or positive. Nonetheless, nonlinear physical relationships have been observed by other researchers such as the cubic relationship between energy consumption and exterior temperature identified in small residential buildings. [52]. In contrast, the linear trends also may be a result of the relatively large buildings considered here (i.e. > 100,000*ft*^2^) where large thermal masses may dampen the effect of weather changes. Therefore, additional work in this area is required to address the complex physics and statistical relationships among the relevant variables.

**Fig 12:**
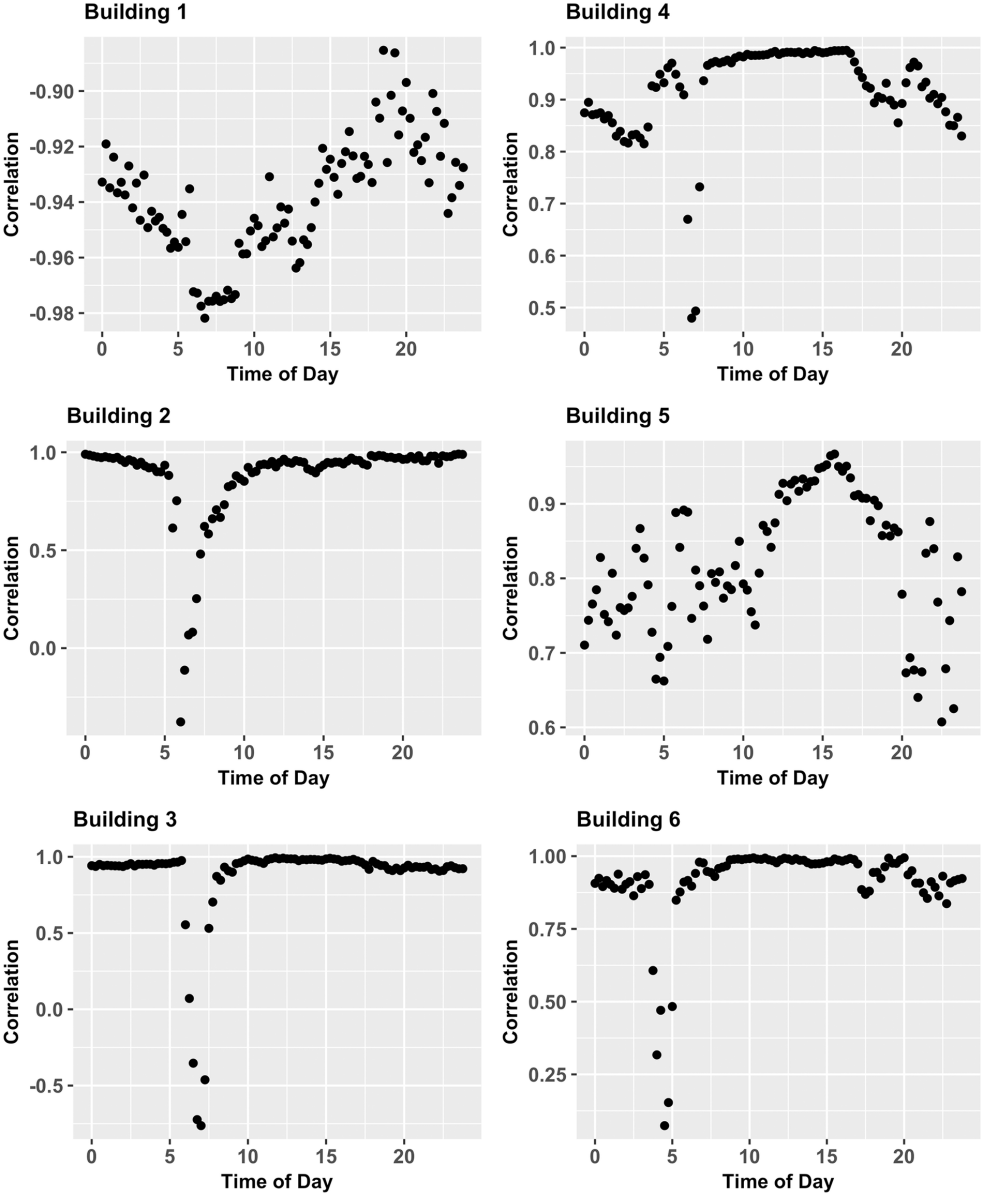
TSAOV method of temperature and electricity consumption. Correlations of time-specific-averaged-ordered temperature and the corresponding averaged electricity consumption for all six buildings.

The overall results are shown in Fig 12. These values are greatly improved when compared to the previous analysis. For many of the buildings, the values exceed a magnitude of 0.9 and report plots with less variability throughout the day. Building 1 specifically shows strong correlation values ranging from -0.88 to -0.98, while buildings 2, 3, 4, and 6 have values pushing the upper limit of 1 throughout the data. These significant improvements in correlation values confirm the strong relationship expected between weather and building electricity consumption. Therefore, this analysis shows that minimizing the random plug load and occupancy, which is not associated with temperature, through grouped averaging is crucial in uncovering the true relationship between HVAC building electricity usage and exterior temperature.

Additional aspects of the later method include the steep dips in correlation. Seen in buildings 2, 3, 4, and 6, they again indicate large scheduled systems which do not necessarily depend upon temperature and therefore interfere with the correlation despite the averaging technique. Furthermore, these four buildings are located about 1,500 miles apart and share a similar characteristic despite differing climates and locations. Another feature of this observation is the curved return to high correlation values after the dip. These curves may indicate the rate at which a building returns to a steady operation during occupied hours when related to temperature.

To compare the correlations between both analyses in a more simplified manner, Table 6 displays the minimum, maximum, mean, and standard deviation among all *n* (96) correlations for each method. Note the distinct difference between the mean values of each analysis. Magnitudes of the mean values range from 0.82 to 0.94 for the TSAOV method, while the simpler time-specific method has correlation mean values ranging from magnitudes of 0.05-0.49. Considering these differences the TSAOV method presents an important strategy to adequately minimize occupancy and plug load from electricity and temperature correlations. Given this ability to deduce high correlations, the TSAOV method can now be further used to determine the correct response of buildings to temperature.

**Table 6:** Statistics of correlations between electricity and temperature from time-specific analyses. The left four columns provide values for the TSAOV analysis, while the right four display the original time-specific analysis.

Building	TSAOV Correlation				Time-Specific Correlation			
	Min.	Max.	Mean	S.D.	Min.	Max.	Mean	S.D.
Building 1	-0.98	-0.89	-0.94	0.02	-0.66	-0.40	-0.49	0.07
Building 2	-0.38	0.99	0.87	0.24	-0.13	0.60	0.32	0.18
Building 3	-0.76	0.99	0.87	0.32	-0.21	0.45	0.33	0.15
Building 4	0.48	0.99	0.92	0.09	-0.05	0.50	0.26	0.14
Building 5	0.61	0.97	0.82	0.09	0.16	0.57	0.29	0.12
Building 6	0.07	0.99	0.91	0.16	-0.26	0.31	0.05	0.15

## Conclusions

An analysis of building energy usage of multiple buildings has provided insight on data cleaning issues, building similarities and differences, relationships between weather and electricity data, and led to the development of the TSAOV method. For accurate analysis, data cleaning must include the identification and mitigation of anomalies such as outliers, missing data, and energy shifts. Determining these erroneous data points gives insights, such as faulty meter recording, and provides a means to quickly and automatically inform an owner about a potential costly billing mistake. This paper has also shown the utility of analyzing multiple buildings and performing a small population analysis. Buildings of different purposes and climates show varying usage characteristics such as baseload, daily operation, and energy use intensity(EUI). Larger population studies of building electricity consumption may lead to further insights, such as detecting unusually high baseload consumption compared to buildings of similar climate, type, and operation.

The weather data sources, NOAA and GIS, were assessed for differences, similarities, and the quality of each. The NOAA and GIS data had strong correlations, although San Jose’s (*r* = 0.887) comparison was weaker than Richardon’s (*r* = 0.995). One should use GIS data if available, especially in locations where climate variability is large, such as the San Francisco Bay Area. However, in correlation analyses and most notably in the electric/non-electric HVAC analysis, both NOAA and GIS resulted in similar findings, indicating the use of either in future analyses is satisfactory. The largest difference between the two datasets however is price and the inclusion of irradiance: NOAA is entirely free and open sourced, while GIS is readily available and includes irradiance, but must be purchased.

Weather to electricity consumption correlations were also studied at length with various analysis strategies. Weather is known to impact the electricity consumption of buildings, therefore significant correlations were expected between consumption and electricity usage. To explore these relationships, correlations were computed in a variety of ways, where only one of the analyses showed strong linear correlation coefficients. Data was correlated between electricity consumption, temperature, and irradiance by the following: directly, accounting for heating/cooling differences, the time-specific method, and the TSAOV method. When omitting the time-specific analyses, the various strategies only resulted in correlation coefficients as high as 0.67, followed by 0.62, 0.60, 0.58 and falling rapidly. Although these few correlations could be considered strong, their methods did not hold considering all six buildings. In fact, the highest correlations were mostly found in the first analysis, directly comparing weather and consumption. This indicated that direct correlations may not have been a result of causation, as non-electric HVAC operations should not combine to lead to higher overall correlations. Therefore, the low correlations found throughout this early data analysis suggest that the correlations are critically hindered by other components of the building consumption, such as the following: 1) non-electric HVAC systems were present and/or the heating/cooling was provided via another building or a district energy configuration; 2) the building had a large thermal mass slowing responses to ambient outdoor temperature; 3) plug load use in the building had no relationship to outside temperature; and 4) building set point temperatures changed in occupied and unoccupied states. Consequently, analyses not accounting for these components (yet still show high direct correlations) are likely coincidental and are not a result of causation due to exterior temperature.

To mitigate these issues the TSAOV method was implemented. This analysis averaged 30+ day groups of most similar temperature values and computed the correlations across the groups. This strategy successfully minimizes the interfering occupancy and plug loads and returned significantly higher correlation values among all six buildings. Based upon this strong temperature-electricity relationship, models can now determine a robust linear coefficient between electricity and temperature from which to predict energy usage with first-order accuracy. Additionally, uncovering this relationship can lead models to disaggregate the temperature component of building energy and subsequently uncover the tendencies of occupancy and plug load, which have previously hindered data analyses.

Together these analyses provide useful information that can lead to the diagnosis of building characteristics and operational efficiencies. As long as a building owner has sufficient access to the building’s electricity consumption data, the data can be ingested into a virtual energy audit tool and insights uncovered within minutes, unlike traditional audits which can take weeks to months. Such a virtual energy audit tool might present results in an easily digestible manner such that the building owner, with limited expertise, can simply understand the findings and recommendations, with additional detail provided to an energy audit team or building energy manager. Additionally, once characteristics about the building and its operation are revealed, results can be compared to a large population of buildings with similar attributes.

Although the data-driven analyses presented here provide many insights, limitations do exist. In particular, this data-driven approach requires the availability of large datasets. In this study datasets of two years at 15-minute intervals for electricity consumption, along with weather datasets, were necessary to achieve statistically significant results. Two years is required because the analyses demand multiple layers of subsetting, and with each subset the data become smaller. Consequently, large amounts of data are necessary to accurately diagnose building characteristics and operational inefficiencies. The 15-minute resolution is required because such resolution captures distinct events within the building and is the most common interval of newly installed smart meters. Of course, many events may occur between each 15-minute interval, such as a piece of equipment turning on while another turns off, and the behavior of each may be lost within the interval. Higher resolution data (e.g. minute or subminute intervals) would provide opportunities for additional insights.

This study was an initial exploration into using a virtual energy audit tool to diagnose building characteristics and operational inefficiencies. Future work includes the additional development of methods, such as TSAOV, to fully uncover the physics-based and non-linear relationships and its impact on results. The TSAOV method can also be further implemented to fully assess the thermal lag or effect of thermal mass in buildings. Future studies also may examine how to diagnose buildings systems and the presence of large equipment determining the size of such systems/equipment and their relative role and importance in the building. Further research is also needed to explore the relative impact of irradiance on building energy consumption, particularly its impact against and with temperature. Finally, an analysis combining insights on energy dependence on temperature, irradiance, lighting systems, non-HVAC equipment, and occupancy induced loads can lead to the successful disaggregation of total building energy consumption. Disaggregating the various loads of a building from analyzing the total load without the need for additional sensoring or sub-metering, can be an incredibly powerful tool. Additionally, one might convert these virtual insights into actionable energy savings opportunities, identifying the potential return-on-investment of various audit possibilities.
